# Recent Advances in Artificial Intelligence to Improve Immunotherapy and the Use of Digital Twins to Identify Prognosis of Patients with Solid Tumors

**DOI:** 10.3390/ijms252111588

**Published:** 2024-10-29

**Authors:** Laura D’Orsi, Biagio Capasso, Giuseppe Lamacchia, Paolo Pizzichini, Sergio Ferranti, Andrea Liverani, Costantino Fontana, Simona Panunzi, Andrea De Gaetano, Elena Lo Presti

**Affiliations:** 1 National Research Council of Italy, Institute for Systems Analysis and Computer Science “A. Ruberti”, BioMatLab, Via dei Taurini, 19, 00185 Rome, RM, Italy; laura.dorsi@biomatematica.it (L.D.); simona.panunzi@biomatematica.it (S.P.); andrea.degaetano@irib.cnr.it (A.D.G.); 2 Department of General Surgery, Policlinico Militare di Roma “Celio”, Piazza Celimontana, 50, 00184 Rome, RM, Italy; biagio.capasso@marina.difesa.it (B.C.); sergio.ferranti@esercito.difesa.it (S.F.); 3 General Surgery Unit, Regina Apostolorum Hospital, Via S. Francesco d’Assisi, 50, 00041 Albano Laziale, RM, Italy; giuseppe.lamacchia@reginaapostolorum.it (G.L.); andrea.liverani@reginaapostolorum.it (A.L.); 4 Department of Intensive Care Unit, Policlinico Militare di Roma “Celio”, Piazza Celimontana, 50, 00184 Rome, RM, Italy; pizzichinipaolo@gmail.com (P.P.); costantino.fontana@esercito.difesa.it (C.F.); 5 National Research Council of Italy, Institute for Biomedical Research and Innovation (CNR-IRIB), Via Ugo La Malfa, 153, 90146 Palermo, PA, Italy; 6 Department of Biomatics, Óbuda University, Bécsi Road 96/B, H-1034 Budapest, Hungary

**Keywords:** artificial intelligence, digital twins, machine learning, immunotherapy, immune checkpoint inhibitor

## Abstract

To date, the public health system has been impacted by the increasing costs of many diagnostic and therapeutic pathways due to limited resources. At the same time, we are constantly seeking to improve these paths through approaches aimed at personalized medicine. To achieve the required levels of diagnostic and therapeutic precision, it is necessary to integrate data from different sources and simulation platforms. Today, artificial intelligence (AI), machine learning (ML), and predictive computer models are more efficient at guiding decisions regarding better therapies and medical procedures. The evolution of these multiparametric and multimodal systems has led to the creation of digital twins (DTs). The goal of our review is to summarize AI applications in discovering new immunotherapies and developing predictive models for more precise immunotherapeutic decision-making. The findings from this literature review highlight that DTs, particularly predictive mathematical models, will be pivotal in advancing healthcare outcomes. Over time, DTs will indeed bring the benefits of diagnostic precision and personalized treatment to a broader spectrum of patients.

## 1. Introduction

Developing a decision support system based on machine learning (ML) is a strategic area of great innovation in the healthcare sector. Indeed, with developments in artificial intelligence (AI) and ML, predictive computer models are more efficient at guiding decisions for better therapies and medical procedures. Recently, digital twins (DTs) have been described as in silico models of diseases or patients which represent a way to evaluate the efficacy of drugs or medical devices, in addition to performing human clinical studies [1].

The push toward systems that are ever closer to the characteristics of the patient derives from the fact that randomized clinical trials are complex and expensive and can expose large populations of patients to unproven and therefore unsuitable therapies for their pathology. Although randomized clinical trials are the gold standard for evaluating the efficacy and safety of new drugs, there is a clear difficulty in patient selection and monitoring, which contributes to significant failure rates [2].

In healthcare, a DT can be a virtual replica of a particular patient, or part of a patient, and thus reflects the patient’s unique genetic or tissue microenvironment composition, or it can be a simulated 3D model showing a patient’s physiological characteristics [3]. Ideally, the development of DTs aims to offer personalized healthcare to individual patients, such as in relation to their response to immunotherapy.

Indeed, recent advancements in immunotherapy for solid tumors are focused on several innovative approaches. One significant development is chimeric antigen receptor (CAR)-T-cell therapy, which has been successful in blood cancers but faces challenges in solid tumors due to tumor microenvironment (TME) immunosuppression. Adoptive cell therapy using tumor-infiltrating lymphocytes (TILs) has shown promising efficacy, particularly in the treatment of advanced solid tumors like melanoma. Other advances include new immune checkpoint targets, beyond PD-1/PD-L1 and CTLA-4, and efforts to reprogram the metabolic pathways of immune cells to make them more effective in combating tumors [4].

There are several benefits to the development of DTs apart from those already mentioned. Health services can be more proactive because predictive algorithms can give answers in real time. DTs potentially have the power to detect anomalies and assess risks, supported by mathematical models that predict the development of a disease or when it will no longer be symptomatic; therefore, the information provided by DTs in the future could help doctors determine what type of early intervention to use on a patient.

AI is also developing in relation to clinical decision-making processes. This area of application arose from the observation of a high frequency of use of antibiotics, which proved inappropriate in neutropenic patients with blood infections, even though the guidelines now seem to be respected by the majority of healthcare professionals [5]. The consequences of this inappropriate use of antibiotics include a greater selection of resistant bacterial strains and avoidable toxicity that can increase mortality.

This kind of approach, as we will see in this review, is based on the use of data retrieved directly from electronic health records (EHRs). This data source is believed to belong to the omics data category, which allows for the development of in silico models [6]. For these reasons, in silico simulations (DTs) are considered an approach that integrates classical clinical studies into the evaluation of experimental drugs and medical devices, the optimization of therapeutic molecules, and the determination of pharmacokinetic (PK) and pharmacodynamic (PD) properties of drugs [7]. The power of AI allows for the integration of huge multi-omics datasets (from immunology to molecular biology and genetics), together with clinical information stored in hospitals’ electronic storage. In addition, the use of EHRs improves patient outcomes [8]. Indeed, AI models can also assign risk scores to facilitate the transfer of high-risk patients to intensive care units or to predict in-hospital mortality, length of stay, and sepsis management (clinical decision-making systems) [6].

Warren McCullough and Walter Pitts were the pioneers in artificial intelligence (AI), introducing the first mathematical model of a neural network in their paper “A Logical Calculus of Ideas Immanent in Nervous Activity”. A few years later, in 1950, Alan Turing expanded on the concept by discussing “thinking machines”, and AI gained its formal recognition in 1956 at the Dartmouth workshop. Thus, AI is broadly defined as a collection of theories, algorithms, and techniques from fields like mathematics, statistics, and computational neurobiology aimed at simulating human thinking [9,10,11].

There are various techniques with which AI can be applied, for example ML, which allows for the acquisition, detection, and learning of patterns of relevant information from large amounts of data through a set of methods and procedures. One of the major features that makes ML an attractive tool for diagnostics and prognostics in medicine and biology is the ability to learn from training data, generalize from historical data, and perform operations without being explicitly programmed.

One of the fields of ML research is so-called deep learning. This type of ML is based on the use of artificial neural networks (ANNs), which are mathematical models that attempt to reproduce the behavior of human brain neuron networks [12].

Exactly as in the human brain, in order to allow an ANN to function correctly, an initial training phase is necessary. During this phase, the ANN learns how to behave with the objective of making decisions autonomously, with minimal human intervention. A specific dataset used in the training phase is the training set. Another dataset, the validation set, is used to evaluate the quality of the training, while the test set is used to evaluate the success of the training.

**Figure 1:**
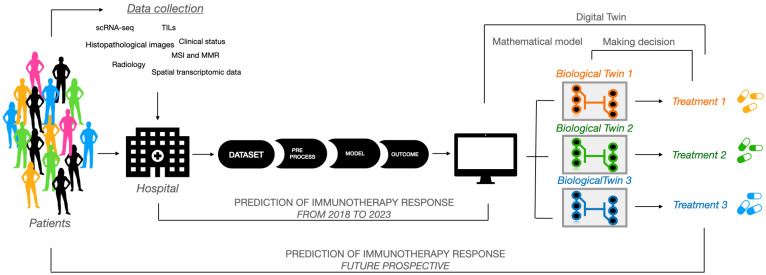
The applications of artificial intelligence in clinical decision support systems. Starting from a pool of heterogeneous data from a large group of patients, such as methylated DNA analyses, scRNA-seq, and image analyses (i.e., histological and radiological), it is possible to divide patients into clusters using AI tools. Each patient cluster will be identified with a digital twin that will support and be supported by a biological twin which, through in vitro assays, will allow for the testing of pharmacological treatments better suited to the patient’s specific conditions.

This type of ML technique is often used to train predictive mathematical models that allow for the identification of the probability of certain future outcomes based on previously collected data [13]. ML algorithms, neural networks, predictive models, and AI in the wider sense can be considered DTs of specific subjects, organs, and biological systems, of which they represent an in silico copy, an “avatar” (Figure 1). 

We focus on the utility of AI models in relation to emergency clinical states. We will therefore analyze pathologies requiring immediate intervention, especially in the oncological field, for which AI is a useful tool in the decision-making phases. Although DTs and ML are of recent biomedical application, there has been an exponential increase in the relevant literature, which prevents us from citing the literature in detail. Furthermore, in this review, we will try to summarize and compare some of the most recent and salient observations of the application of ML and DTs in clinical cases related to urgency and emergency.

## 2. Study Design

This study aims to explore the use of artificial intelligence technology in emergency medicine and discuss its potential future applications to assist clinicians in selecting immunotherapies quickly and effectively. A literature search for English-language publications was conducted using MEDLINE/PubMed (https://pubmed.ncbi.nlm.nih.gov/) and Google Scholar. Although no time restrictions were applied to the search strategy, allof the retrieved publications span the period from 2018 to 2023. The search terms used were “Digital Twins”, “Clinical Decision-Making”, “Artificial Intelligence”, and “Immunotherapy”, combined using “AND”. Studies from any country were considered. However, given the popularity of the topic and the large volume of material available, we focused our review specifically on AI applications in clinical decision-making models, particularly those involving the creation of predictive computer models. We focused solely on solid tumors, with an emphasis on metastases, as they are often associated with urgent or emergency conditions.

We herein describe the use of immunotherapies, focusing on how AI can enhance their effectiveness. Finally, we highlight the use of digital twins as a highly relevant experimental model that generates data for AI applications, thereby improving patient diagnosis and prognosis. For an explanation of our strategy, please see the PRISMA diagram (Preferred Reporting Items for Systematic Reviews and Meta-Analysis) (Supplementary Figure S1).

## 3. Metastasis: The Critical Turning Point in Cancer Progression

Metastases are the leading cause of death in patients because cancer cells spread from the site of the primary tumor to other parts of the body, becoming an obstacle to eliminating the cancer. It remains the most critical phase of tumor progression and a primary challenge in treating cancer. With the approval of additional first- and second-line systemic treatments, the therapeutic landscape has grown rapidly in the last two years, in some cases also resulting in targeted therapies. A greater availability of systemic therapies determines an increased chance of longer survival but raises a problem regarding the choice of therapeutic combinations and sequential treatment. Moreover, deep learning tools can analyze, for instance, whole-slide images (WSIs) to identify lymph nodes and tumor regions and reveal the tumor-area-to-MLN-area ratio (T/MLN) [14]. These findings indicate that deep learning models may be able to assist pathologists not only in detecting lymph nodes with metastases but also in speeding up decision-making processes.

### 3.1. Metastases and Digitizable Therapeutic Interventions

Many types of cancer metastasize to bones, and prostate and breast cancers are the most frequent primary tumors with bone metastases [15]. In addition, about 10–20% of patients with osteosarcoma develop lung metastases [16]. According to the anatomical–mechanical hypothesis, through lymphatic diffusion, for example, lung and breast cancer metastasizes to the axillary lymph nodes. Similarly, due to the portal venous system, many gastrointestinal organs metastasize to the liver. Colorectal, ovarian, and stomach cancers often metastasize to the abdominal cavity, and lung cancer metastasizes to the chest. According to the “seed and soil” hypothesis, some cancers may prefer to migrate to organs with a similar environment. For example, several histological subtypes in lung cancer were shown to metastasize to the liver (small-cell lung cancer) and nervous system, containing neuroendocrine cells, while adenocarcinomas often metastasize to bones. Or, finally, the dissemination could be due to “metastatic speciation”, i.e., the acquisition of genetic and epigenetic variations in distant localities that allow for metastatic expansion [17]. 

The treatment of metastasis requires tools to assist in shared decision-making, especially thanks to the introduction of immunotherapeutic combinations. Indeed, for metastatic patients, immunotherapy has become the first line of intervention [18], and thus, further combinations are desirable.

### 3.2. Immune Checkpoint Blockade Immunotherapy

CTLA-4 (cytotoxic T lymphocyte-associated antigen 4) and programmed cell death protein-1 (PD-1) receptors are normally expressed on T cells and bind to B7 and programmed death ligand-1 (PD-L1) receptors on APCs (antigen-presenting cells), respectively, to regulate the immune system. Based on these interactions, anti-checkpoint antibodies that prevent these interactions have been developed [19]. Therefore, immune checkpoint blockades (ICIs) are monoclonal antibodies that enhance the anti-tumor activity of T cells by blocking the CTLA-4/B7 or PD-1/PD-L1 immune checkpoint pathways, thereby activating the immune system [20].

ICIs are clinically effective not only because they directly target tumor cells expressing PD-L1 proteins but also because the high mutational burden of tumor cells appears to increase their expression of PDL-1 [21]. Finally, ICIs improve the immune system’s ability to detect and eliminate neoantigens in tumor cells resulting from somatic mutations capable of binding with the major histocompatibility complex (MHC) for presentation to T cells [22]. 

In 2021, ICIs had been approved for 18 cancer types [19], and ICIs targeting several immune checkpoints in combination with other ICIs are still under study. Advances in this field stem from the understanding that it is more beneficial to overcome immune suppression in the tumor microenvironment than to trigger immune activation, thus ushering in the era of cancer immunotherapy [23]. 

The decision about which immunotherapy to administer, or whether a combined approach (chemotherapy and ICI) is justified, can be guided by an algorithm that rationally maximizes disease control, reduces side effects, and minimizes costs. A rational approach could therefore also predict the adverse effects of therapy and recognize patients most at risk of toxicity so that they can be adequately supervised and treated.

**Figure 2:**
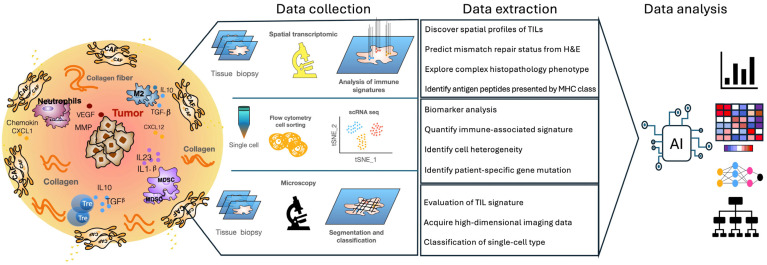
The analysis and identification of immune signatures through AI technology. Through machine learning algorithms, AI analyzes a vast amount of data, looking for correlations between different types of immune cells, their gene and protein expressions, and the observed immune responses. Additionally, AI uses neural networks to identify complex patterns in the interactions between immune cells and pathogens or tumors, and to detect specific signatures such as T-cell activation, the presence of NK cells, and antibody responses. AI allows these results to be visualized through graphs and heat maps, flow diagrams, and network graphs.

To further enhance the effectiveness of ICIs, understanding the tumor microenvironment, including factors such as tumor-infiltrating lymphocytes (TILs) and tertiary lymphoid structures (TLSs), is crucial. For this reason, it is necessary to integrate information from histopathological features, imaging omics features, genomics, and scRNA-seqs of the immune system (this approach is commonly used in AI analysis, as described in Section 4). Understanding the tumor microenvironment also involves evaluating the density of tumor-infiltrating lymphocytes (TILs) within the tumor, which is regarded as a potential predictor of ICI response. Indeed, TIL density is a powerful positive prognostic indicator for some tumor types, despite ICI therapy. Indeed, a parameter called Immunoscore, which predicts the quantification of CD8+ T cells in the center and periphery of a tumor, is a strong predictor of overall survival that can complement traditional tumor–node–metastasis staging or microsatellite instability (MSI) status in colorectal cancer (CRC) [24,25]. In particular, single-cell sequencing demonstrated that CD4+ memory T cells are also enriched in ICI 180-responsive human melanomas. In the context of anti-PD1 therapy, TIL density measured by immunohistochemical (IHC) tests at the invasive margin of a tumor, relative to central infiltration, has been reported to be strongly associated with anti-PD1 response. We therefore find ourselves in the field of omics data, whose degree of complexity requires AI tools for their analysis [26] (Figure 2). 

Not only TILs but also TLSs can provide useful information for predictive efficacy or immunotherapy [27]. TLSs have been identified in several human tumors and described not only for their cellular constituents but also for their location within the tumor in both primary and metastatic lesions [28]. Their heterogeneity could influence the efficacy of the anti-tumor immune response and patient outcome [29]. 

Therefore, it is believed that TLSs should be considered not only indicators of an active immune response but also immunoregulators of the anti-tumor response. Mature TLSs exhibit evidence for the formation of germinal centers in colon cancer and in lung squamous cell carcinoma [30,31], and oligoclonal B-cell responses have previously been detected in cutaneous melanoma and metastases [29], indicating an active humoral anti-tumor response within TLSs that is driven by B cells. 

Given the vast and detailed number of papers on ICB therapy, we have attempted to recapitulate only some of the more recent and crucial observations on the topic. 

## 4. AI Applications in Cancer Immunotherapy

AI can be used for three main aims in the immunotherapy field: (1) identifying novel neoantigens, (2) designing antibodies, and (3) predicting immunotherapy effects (Table 1).

**Figure 3:**
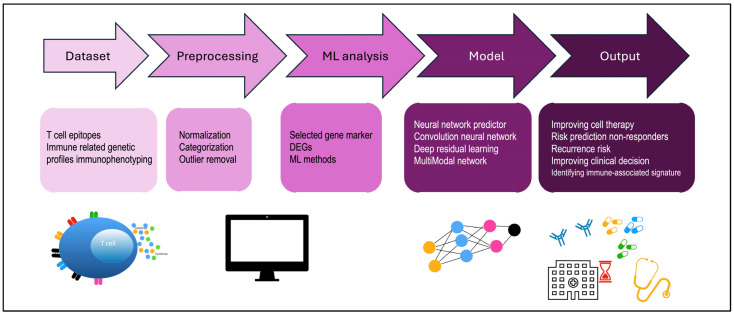
A step-by-step illustration of the application of AI methodologies employed, and how they integrate with immune-associated signatures and impact patient outcomes. AI neural networks can be used to predict immune responses by simulating immune reactions to tumors. This leads to better clinical decisions, such as the use of antibodies and immune checkpoints (like PD-1/PD-L1), or to preventing the recurrence of the disease. Characterizing the patient’s immune signature through AI allows for the development of new antibody therapies. Machine learning (ML); differentially expressed genes (DEGs).

1. Neoantigens are pivotal to the development of immunotherapies against cancer because they are proteins that exhibit immunologically active mutations and, therefore, are useful for inducing immune system response [32]. These rare mutations are called immunogenic. A key challenge for CAR-T-based immunotherapies (which involve the transfer of T-cell receptors (TCRs) into the recipient patient’s T cells) is the recognition of specific target antigens that prevent the engineered TCR-T cells from attacking the antigens expressed on healthy tissues [33]. Therefore, the identification of a wider variety of mutations such as gene fusion, alternative splicing, mutational frame shifts, or the presence of endogenous retroviruses could improve the design of therapeutic cancer vaccines and, consequently, evaluations of the efficacy of immunotherapy. From 2016 to 2021, 19 different models were developed with different approaches and datasets in independent studies, as reported in Li et al. [18]. These approaches aim to improve epitope–MHC interactions, and the accurate prediction of MHC binding or presentation could also contribute to neoantigen identification by considering post-translational peptide modifications and immunogenicity [34].
2. The immune system protects our body from viral and bacterial pathogens, as well as from neoplastic episodes that occur during our lives. Tumors deploy immunological evasion mechanisms; so, to help the immune system fight cancer, immunotherapy has been developed as a treatment that provides the protection and strengthening of the immune response. AI-based methods for antibody design are aimed at evaluating three different roles: target binding prediction, with five independent studies [35,36,37,38,39]; antibody structure prediction, with five different models predicting relative distances and orientations of the antibody variable fragment (Fv) region, orientations of antibody CDRH3 regions, structures of antibody CDR loops, or VH domains of antibodies [40,41,42,43,44]; and pharmaceutical properties, with two published papers on the same model (DeepSCM) focused on forecasting solubility, viscosity, and biophysical properties [45,46], as well as three studies on antibody humanness evaluation and the evaluation of the nativeness of antibody candidates [47,48,49]. Indeed, one immunotherapeutic strategy involves the design of monoclonal antibodies that recognize the antigen in a specific way and can also be used for drug delivery. An antibody–drug conjugate (ADC) is characterized by the union between a monoclonal antibody (mAb) and a cytotoxic drug linked by a covalent bond. To date, eleven ADCs and two bispecific antibodies, blinatumomab and amivantamab, have been approved for the treatment of cancer [50]. Therefore, AI has become a tool for predicting antibody structures with a number of desirable qualities, including the ease with which it is produced, its stability in storage processes, its ease of administration, and its effectiveness in patients [51]. For example, McDermott et al. used two new computational platforms [52], NCI’s CellMiner Cross database and RADR^®^ AI and ML platform (from Lantern Pharma Inc.), to evaluate potential new targets for the acylfulvene-derived drugs LP-100 (Irofulven) and LP-184. Data from CellMinerCDB predicted that LP-184 and LP-100 would be effective in ultra-rare and fatal childhood cancers and atypical teratoid rhabdoid tumors (ATRTs), characterized by chromatin remodeling deficiencies. Subsequently, Lantern’s RADR^®^ AI and ML platform was useful for building an in silico model to test whether LP-184 would be effective in atypical teratoid rhabdoid tumor (ATRT) patients, a rare and aggressive tumor of the central nervous system [52]. In the same way, they rapidly developed novel cryptophycin ADCs, which are an exciting class of potent and highly targeted drug candidates for breast cancer [53]. Despite significant improvements in anticancer immunotherapy for different types of tumors, positive treatment results have not been observed in all patients.
3. One major challenge is to understand the mechanisms of immune resistance and to identify potential predictors of effective responses. Immune checkpoint blockades (ICBs) have rapidly revolutionized treatment plans for various types of cancer. Numerous single treatments or combinations of ICB have provided more options for patients after approval by the US Food and Drug Administration. However, primary and acquired drug resistance does not allow most patients to benefit from these immunotherapies. Therefore, the prediction of the therapeutic effect based on the use of AI increases the chances of success of anti-tumor immunotherapy because it is based on objective data, such as the definition of predictive scores of immunotherapy, as formulated by Angell et al. almost ten years ago [54]. With the ability to quantitatively analyze the expression of PDL-1 on melanoma tumor cells and thus obtain a score, some studies have used AI to predict PD-L1 expression using a supervised ML algorithm (random classifier of forests) [55]. In general, incorporated histopathological images and clinical information was used to predict tumor mutational burden (TMB) and MSI in non-small-cell lung cancer (NSLC) [56,57,58] and CRC [59]. However, the idea that PD-L1 expression was a specific biomarker to predict immunotherapy was recently revised, as good efficacy was observed even in cancer patients with low PD-L1 expression [60]. AI-based prediction of clinical outcomes in immunotherapy is employed in several types of studies on different tumor tissues. For NSCLC, there is a predictive model based on PD-L1 expression (tumor proportion score (TPS)) in which an AI-assisted scoring system helped pathologists, serving as a scoring tool [61]. In this case, as in many others, pathologists validated the results obtained from the AI tool [62]. Furthermore, the DL convolutional neural network through tumor feature extraction and selection algorithms achieved 90% accuracy in detecting melanoma and breast cancer lesions. This information is critical for determining which patients are best suited to receive ICBs and, at the same time, assessing the risk of immune-related adverse events (irAEs) prior to treatment. irAEs are considered the most toxic reactions associated with checkpoint inhibitors as they are involved in disrupting immune homeostasis [63]. The tissues most affected by irAEs are mainly the skin, gastrointestinal tract, and endocrine organs, but they can also affect other tissues of the body. In the most extreme cases, irAEs can lead to patient death [64]. Since a single biomarker is not sufficient to predict the efficacy of immunotherapy, AI is needed to develop a predictive model that includes multiple parameters related to tumor–host interactions [26,65], and related adverse events can also be predicted, as demonstrated in recent publications using ML methods [66]. Deep learning techniques, therefore, have applications in identifying genes, phenotypes, and their relationships based on clinical status and patient response [67]. All of this mass of information, for example from H&E, qPCR (quantitative real-time PCR), IHC, and NGS (next-generation sequencing), has become increasingly complex because it concerns the genetics, transcriptomics, and proteomics of a complex of heterogeneous cells. TMB, which consists of the total number of somatic coding mutations in a tumor, has been emerging among the predictive biomarkers for immunotherapy response in cancer patients [68]. In fact, whole-exome sequencing (WES) is considered the gold standard for having a complete measurement of TMB. In colorectal and stomach cancer, the prediction of MSI and MMR is primarily achieved by analyzing Hematoxylin and Eosin (H&E) images (H&E is a widely used technique in pathology and histology to visualize tissue structure under a microscope) using various machine learning methods, such as convolutional neural networks or ShuffleNet [69,70,71]. The large amount of available data allows for model validation and ensures high accuracy in the results. F. Xie et al. [72] developed a predictive model integrating TMB, MSI, and somatic copy number variation (CNV) across different tumor types to find differences between patients who have infiltrated immune cells (“hot” tumor) and those with little infiltration (“cold” tumor). The model proposed that “hot” immune patients had a better prognosis because they were more responsive to immunotherapy. Moreover, it is possible to integrate WES and RNA sequences, showing that the response rates of metastatic melanoma patients to anti-CTLA4 was associated with TMB and cytolytic markers [73]. These analyses also allowed J. Xie et al. to demonstrate that in patients with triple-negative breast cancer, there is a correlation of the expression and variant levels of platelet-related genes with the prognosis and immunotherapy response [74]. These relationships are also applicable for vaccine development; for example, it is possible to combine exome sequencing, transcriptome sequencing, and mass spectrometry to discover immunogenic mutant peptides with MHC-specificity [75]. Indeed, Mo et al. have already implemented a platform for high-throughput screening to observe interactions between immune cells and cancer cells of the same patients, assessing viability and cell growth phenotypes. This type of study has also identified three potential antagonists for increasing immune activity [76]. The analysis of the tumor microenvironment (TME) is still a main goal in many studies, and our research group has pioneered the understanding and characterization of anti-tumor mechanisms of γδ T cells in solid tumors [77,78]. In fact, the presence of TILs, as along with higher levels of some cytokines, chemokines, and activated cytotoxic T cells, is correlated with superior clinical outcomes, especially in the efficacy of CAR-T-cell therapy. It has been noted that pretreatment of the TME was used to predict the safety of CAR-T cells, such as the incidence of neurotoxicity [79]. A precise description of the TME of colon, breast, lung, and pancreatic solid tumors can be accurately estimated with ML and DL approaches by integrating scRNA-seq (single-cell RNA sequencing) and imaging data in a clinical setting [80], or with methylation data [81]. In regard to deciphering TMEs, we found four independent studies focused on the prediction of the spatial patterns of tumor-infiltrating lymphocytes (TILs) by using H&E images and bulk RNA-seq or spatial transcriptome analysis [82,83,84,85]. Thus, today, we can find an accurate reconstruction of the TME using bulk RNA-seq [79] and spatial transcriptomics [80] to understand the organization and molecular correlation of TILs thanks to ML algorithms (Figure 3). Regarding the prediction of immunotherapy response, Kong et al. [86] analyzed transcriptomic data from three different solid tumors, while Vanguri et al. [87] assessed the response of NSCLC patients treated with ICBs by integrating histopathological images and genomic data. These analyses emphasized the importance of integrating multiple sources of patient information to effectively validate the results and to predict the response to Abs inhibitors across 16 different cancer types [88].

**Table 1:** A summary of publications regarding the application of AI in improving immunotherapy, both in terms of efficacy prediction (based on structure and properties) and response prediction.

Medical Field	Identifying Novel Neoantigens				
Time	Biomarker	Methods	Source	Outcome	Models
From 2016 to 2021	MHC peptide I and II class	Deep learning; recurrent neural network; neural network; convolutional neural network; machine learning; natural language processing	Mass spectrometry datasets; Immune Epitope Database (IEDB); SYFPEITHI database; RNA-seq data	Improving epitope–MHC interactions; MHC stability; immunogenicity; TCR binding; prediction of paired α/β TCR	(18 distinct studies) model EDGE, DeepHLApan, NMER, and NetMHC-4.0; NetMHCpan-4.0; MHCflurry; MHCflurry-2.0; Neonmhc2; Neopesee; pMTnet; ForestMHC; PRIME; MARIA; MHCSeqNet; HLAthena; NetTCR-2.0; NetMHCPan-4.1; NetMHCIIpan-4.0 [18]
**Medical Field**	**Designing Antibodies**				
**Time**	**Target**	**Methods**	**Source**	**Outcome**	**Models**
From 2019 to 2022	CDRH3 regions and trastuzumab; CTLA-4 and PD-1 Abs; emibetuzumab.	Deep learning; convolutional neural network; long short-term memory;	CDR-H3 sequences public datasets	Improving target binding	(5 distinct studies, only 1 has a name) Ens-Grad [35,36,37,38,39]
	CDRH3 regions; CDR loops; or VH domains of antibodies.	Convolutional neural network; deep residual learning; ReNet	CDR-H3 sequences	Antibody structure prediction	(5 distinct studies) DeepH3; DeepAb; DeepSCAb; ABlooper; NanoNet [40,41,42,43,44]
	Abs solubility; viscosity.	Bi-LSTM network; RoBERTa; convolutional neural network; random forest classifier	Antibody sequences from repertoire sequencing	Forecasting pharmaceutical properties	(5 distinct studies) AbLSTM; BioPhi; solPredict; DeepSCM (used in two independent publications) [45,46,47,48,49]
**Medical Field**	**Predicting Immunotherapy Effects**				
**Time**	**Target**	**Methods**	**Outcome**	**Source**	**Cohort**
From 2018 to 2022	Colorectal cancer and stomach cancer	Convolutional neural network; deep residual learning	Prediction of microsatellite instability	H&E histology from tissue banks	*n* = 94 whole-slide images from *n* = 81 patients [69,70]
	Colorectal cancer	ShuffleNet; MobileNetV2	Prediction of defective DNA mismatch repair and microsatellite instability	H&E histology from MSIDETECT consortium study	*n* = 8836 colorectal tumors (of all stages) [71]
	Cutaneous melanoma	Random forest classifier	Expression level of PD-L1 for precision of PD-L1 scoring	H&E histology	*n* = 69 cutaneous melanomas [55]
	Non-small-cell lung cancer	Deep learning	Tumor prediction score of PD-L1 expression	Whole-slide images	*n* = 173 IHC assay by using 22C3 binding Ab [61]
	Colorectal cancer	Deep learning	Prediction of tumor mutational burden	Histopathological images	*n* = 631 CRC patients in TCGA [59]
	24 cancer types	Machine learning	Prediction of cell composition in TME	Bulk RNA-seq	*n* = 9404 RNA-seq samples [79]
	13 cancer types	Convolutional neural network	Prediction of spatial cell composition in TME	H&E images	*n* = 4759 TCGA subjects [80]
	Breast cancer	Deep residual learning	Prediction of cell composition in TME	H&E image	*n* = 64 patients [81]
	Lung adenocarcinoma	Convolutional neural network	Prediction of spatial cell composition in TME	Spatial transcriptomic data and H&E images	*n* = 21 H&E images [82]
	Melanoma, gastric cancer, and bladder cancer	Machine learning	Prediction of immunotherapy response	Transcriptomic data	*n* = 91 melanoma; *n* = 45 gastric cancer; *n* = 348 bladder cancer [86]
	29 cancer types	MultiModal network		RNA-seq; genomic data	*n* = 8646 The Cancer Genome Atlas samples [69]
	Non-small-cell lung cancer	Multiple-instance LR		Histopathological images; genomic data	*n* = 247 patients [87]
	16 cancer types	Random forest	Prediction of Abs inhibitor response	Genomic and molecular data	*n* = 1479 patients [88]

## 5. AI Supports DT Development

To date, the public health system has been affected by the increasing costs of many diagnostic and therapeutic pathways due to limited resources. At the same time, we are constantly seeking to improve these paths through approaches aimed at personalized medicine. To achieve these levels of diagnostic and therapeutic precision, the integration of data from different sources and simulation platforms is necessary. As we have already said, the application of the computational systems furnished by AI can generate practical solutions to resolve these critical issues. In this scenario, DTs and BTs appear to be simulation systems that are capable of predicting the outcome of individual patients and their responses to therapy, and it seems clear that one twin feeds the other with information. 

However, it is necessary to make a precise distinction between DTs and BTs. In fact, these two concepts are often confused and are not very well defined, even though they are fed by different but complementary data and information sources. A DT is a virtual system of a process used to analyze in detail the performance characteristics of its real counterpart and predict its results. By incorporating multiphysics simulations, data analytics, and ML capabilities, DTs have the advantage of demonstrating the impact of changes in biological processes such as environmental conditions or the occurrence of mutations, improving the quality of the final product or process. Instead, creating a digital biological twin means using a patient’s tissue sample to develop a 3D disease model, such as a tumor cell culture or an organoid. We then monitor this model to track the progression of the patient’s disease and test different drugs at different doses at multiple time points. This process allows us to collect a wide range of data, resulting in a comprehensive multimodal monitoring tool. This system is embedded in a microchip where the fluidic system connects different cellular compartments, keeping the system of cell–cell interactions in a dynamic form. Then, the BT is the result of in vitro models that can identify specific data/parameters and simulate drug response by reproducing a part of the patient to add information to the simulation capabilities of the DT. Thus, AI can integrate the concept of digital twins with information obtained from in vitro models in several (Table 2) ways, by processing data in real time and combining data from in vitro models with patient clinical data, and can virtually test different therapies and dosages before applying them to the patient. In summary, AI can transform data obtained from in vitro models into actionable insights, improving the accuracy and speed of clinical decision-making through integration into the digital twin model.

The DT continually evolves and updates to reflect changes to the physical counterpart of an entire process, creating a closed feedback loop in a virtual environment, feeding on new information that continues to optimize the product and performance at low costs. The data obtained from digital models complement the data sources already available for the analysis, such as retrospective data, patient cohort data (interoperable platforms, wearable devices, sensors, and biomarkers), and personalized patient data. Usually, the first data points are deposited in a (public) database and have already been generated, therefore being retrospective. It must be considered that DT data are dependent on time and variations, unlike personalized patient data which are defined as “punctual” because they are measured following programmed steps. Furthermore, DT data change over time because they are subject to updated and continuous simulations. Therefore, a DT provides continuous analysis and forecasts over time, making it a useful tool for the formulation of mathematical models. One of the most complex examples of a DT is the gut–brain axis (GBA), which has great potential to prevent and treat gastrointestinal disorders and will prospectively allow us to influence behavior by providing us with an effective, continuous interaction and predictive state of the GBA [87]. 

The idea is, therefore, to obtain AI algorithms (for instance, deep and shallow ML, hybrid machine learning, Bayesian inference, mixed-effects models) to model crucial related mechanisms such as immunological response in tumors, which is to date one of the most studied topics in the international scientific panorama. In fact, in the oncology field, apart from problems related to the complexity of neoplastic processes and the heterogeneity of the events involved, mathematical models face several challenges: (1) the adaptation of the observational experimental data to the models, resulting in personalized model parameters, which is necessary because each patient has their own gene mutations, immunological responses, and clinical context; (2) the models use a simplified view of tumor cells, ignoring their spatial position, the composition of the tumor microenvironment, the process of competition for nutrients in the bone marrow and the cell cycle, and other mechanisms concerning more specific cellular biological processes, while other models, such as those for application in radiology or surgery, require many geometric representations; (3) simple mathematical models are obviously limited to very simple statements, while it is necessary to know, for example, whether the immune system responds effectively to immunotherapy during the disease. Thus, while AI is already an established tool for analyzing complex and multimodal data, we emphasize the importance of mathematical models in improving therapeutic decision-making. 

For this reason, it is necessary to equip DTs with multiple mathematical models, with different levels of detail and characteristics, with the aim of answering a specific clinical or research question [88]. 

In oncology, mathematical models integrated into digital twins provide a powerful tool for simulating the complex dynamics of cancer progression and treatment response. By incorporating patient-specific data and modeling the interactions between tumors, therapies, and the immune system, digital twins enable personalized, data-driven clinical decision-making and optimization of cancer treatment strategies. Thus, mathematical models play a crucial role in the creation of digital twins in oncology by simulating and predicting the behavior of tumors and their interactions with therapies. In Table 3, we provide a detailed description of how mathematical models are used to create digital twins.

Furthermore, it is important to note that DT data are structured data, and this is reflected in their dependence on time and interventions. In contrast, personalized patient data are measured in scheduled steps, while DT data change over time because simulations can consider updated patient information with continuous simulations. Overall, a cohort of DTs is calibrated to a target patient at time ti, and each DT simulates the effects of a specific intervention so that each returns the best possible approximation to simulate biologically realistic data during medical procedures, such as drug administration, determining suitability for surgery, etc. 

There are studies that aim to use classical ML techniques to create DTs that are reliable for medical specialists, and for which they do not require in-depth knowledge of AI, and meet the requirements of the Internet of Medical Things (IoMT) in terms of latency and costs [89]. A more concrete attempt has been made for breast cancer, one of the most widespread forms of cancer, for which the feasibility and reliability of DTs in patient monitoring and diagnosis have been demonstrated [90]. An example of a primitive DT is the artificial pancreas, which consists of closed-loop automated control of diabetes mellitus. Indeed, people with type 1 diabetes mellitus face the problem of permanently optimizing glycemic levels without increasing the risk of hypoglycemia. The insulin pump is linked to real-time continuous glucose monitoring and employs mathematical algorithms that connect the pump to the sensor for monitoring, with performance comparable to the normal pancreas [91]. In the context of ML applications for the creation of DTs, mathematical models try to capture the dynamics of biomarkers, the proliferation of tumor cells at a given time t, considering a constant value r, which can be associated with the difference between proliferation and cell death, or the interactions between different cell populations, for example. This kind of approach allows us to answer the question “What would happen if...?” Implementing mathematical models of this type means adapting experimentally observed data to the models, consequently obtaining parameters customized to the type of patient. As we have already described, a simple mathematical model is obviously limited by very brief statements from which, however, we want to draw a more in-depth vision of what would happen if, for example, we administer drugs or how the immune response is influenced during the progression of the disease. Susilo et al. demonstrated that DT parameters inferred from responding and non-responding patients showed potential biological differences that may influence their response to mosunetuzumab, such as tumor size, the degree of T cell infiltration, or tumor cell proliferation, but also showed parameters relating to the activation or cytotoxic mechanisms of immune cells. The model therefore suggests that intratumoral expansion of pre-existing T cells underlies the anti-tumor activity of mosunetuzumab. [92]. This conclusion will allow us to implement DTs for new clinical and laboratory observations, acquiring greater similarity to the reality of the biological mechanism underlying the response to immunotherapies. 

Brain tumors can be diagnosed through segmented MRI imaging. There are brain tumor detection algorithms that are based on the analysis of brightness contrast in MRI scans and electroencephalogram (EEG) recordings. This information can be processed in the DT to improve diagnosis. To date, the algorithm is able to identify MRI images of healthy patients from those with tumors with an accuracy varying from 85% to 96% [93]. Furthermore, the proposed DT platform uses a multimodal dataset (on the patient’s psychological state, metabolic characteristics, immune and epigenetic profiles) implementing various ML methods. The platform also includes health reports. All of this processed information updates the DTs of cancer patients, acting as a platform on which to investigate the best treatment method or helping clinicians make decisions [90]. Moreover, DTs seem to be a revolutionary tool for improving diagnosis, monitoring, and therapy, benefitting patients’ well-being, lowering economic costs, and enabling the prevention of disease progression in Multiple Sclerosis patients [94]. Among the applications of DTs, we found an interesting use for precision medicine in vascular surgery, such as detecting the severity of carotid stenoses from head vibrations in a work that coupled computational mechanics and a computer vision method [95,96,97], but many other applications in healthcare have been reported in the review by Sun et al. [98]. 

## 6. Conclusions

Compared to previous works, this review aims to combine the use of AI to improve clinical practice while highlighting the growing significance of digital twins as an experimental model. We have also recognized that biological twins provide valuable data that can be processed by AI, delivering increasingly rapid and useful information for patient management in emergency situations. In the last 5 years, the number of publications using artificial intelligence methods to leverage image data or scRNA-seq data, combined with clinical information, to create highly accurate prevention strategies has been steadily increasing. We believe that DTs are a resource in the medical field for the resolution of problems such as the precise treatment of diseases, the administration of personalized therapies, etc., as well as clinical decision support systems in urgent–emergency contexts for which the time factor is extremely decisive. In this regard, we are aware that there are other types of immunotherapies, such as CAR-T-cell therapy, cancer vaccines, oncolytic virus therapy, and cytokine therapy, in addition to ICBs, which are applied to cancer patients in emergency conditions, such as metastatic patients or those with chronic autoimmune diseases. The availability of such a wide range of therapies allows for the application of personalized medicine, which we hope can be supported by AI models to facilitate rapid therapeutic decisions based on predictive models developed in digital twins (DTs). This review also shows that mathematical models are at the heart of digital twin technology, especially in the simulation of complex systems, from biological processes to industrial machines. Although still in their early stages in fields such as personalized medicine, the integration of AI, real-time data, and multiscale modeling will drive major advances in the near future. What emerges from the studies summarized in this work is the need to use DTs for predicting the patient’s state of health and the pharmacological efficacy of drugs. These considerations place the DT as a healthcare assistant that stands between future medicine and an everyday medicine which aspires for precision and the administration of personalized therapies.

## Figures and Tables

**Table 2:** How artificial intelligence (AI) can integrate digital twins (DTs) with biological twins (BTs).

Complex Data Analysis	AI Function
In vitro models, such as cell cultures or organoids, generate vast amounts of data (e.g., images, genomic, proteomic, and metabolomic data).	AI can process these data in real time, identifying complex patterns and relationships that may not be evident through traditional analyses.
**Personalized Predictions**	**AI Function**
The digital twin is fed by multimodal data, allowing for simulations of disease progression and predictions of how the patient will respond to various treatments.	AI can combine data from in vitro models with patient clinical data, such as blood tests, diagnostic images, and genomic information.
**Treatment Optimization**	**AI Function**
Integrating information from in vitro models with simulations in the digital twin reduces risks and improves treatment effectiveness.	AI can virtually test different therapies and dosages before applying them to the patient.
**Dynamic Updates**	**AI Function**
Continuous updates on the patient’s health status are provided as well as personalized management of treatment, such as adjusting therapies.	AI enables the digital twin to continuously update itself with new data from both the patient and in vitro models.
**Accelerate Discovery**	**AI Function**
The discovery of therapeutic targets goes hand in hand with the new knowledge gained on solid tumors thanks to the sophisticated technologies available today.	AI can speed up drug discovery processes and treatment optimization by running large-scale simulations and rapidly integrating data from in vitro models.

**Table 3:** How mathematical models help the creation of digital twins in oncology. Mathematical models play a crucial role in the creation of digital twins in oncology by simulating and predicting the behavior of tumors and their interactions with therapies.

Field	Modeling Tumor Growth		
Mathematical models, such as ordinary differential equations (ODEs) and partial differential equations (PDEs), are used to describe the following:	Cell proliferation and death	Tumor microenvironment	Spatial growth patterns
**Field**	**Immune Response Dynamics**		
Mathematical models simulate the interaction between the tumor and the patient’s immune system in specific way:	Immune cell infiltration	Cytokine signaling	Immunotherapy response
**Field**	**Drug Response and Therapy Optimization**		
Models of pharmacokinetics (PK) and pharmacodynamics (PD) are used to simulate how drugs are absorbed, distributed, metabolized, and eliminated in the body. These models help to predict the following:	The effectiveness of chemotherapy or targeted therapies based on drug concentration at the tumor site	Optimal dosing regimens to minimize side effects	Resistance mechanisms that may emerge during treatment
**Field**	**Metastasis Simulation**		
Mathematical models can track how cancer cells migrate from the primary tumor to form metastases in other organs. These models simulate the following:	Cell migration	The process of tumor cells establishing new metastatic sites	Therapy resistance in metastases
**Field**	**Predicting Outcomes and Clinical Decision Support**		
Digital twins built on mathematical models can predict future scenarios, such as the following:	The likelihood of tumor recurrence after surgery or chemotherapy	How long a treatment will remain effective before resistance develops	Which combination of therapies will yield the best outcomes based on the tumor’s specific characteristics

## Data Availability

No new data were created or analyzed in this study.

## Supplementary Materials

The following supporting information can be downloaded at: https://www.mdpi.com/article/10.3390/ijms252111588/s1.

## References

[1] Moingeon P., Chenel M., Rousseau C., Voisin E., Guedj M. (2023). Virtual patients, digital twins and causal disease models: Paving the ground for in silico clinical trials. Drug Discov. Today.

[2] Viceconti M., Henney A., Morley-Fletcher E. (2016). In silico clinical trials: How computer simulation will transform the biomedical industry. Int. J. Clin. Trials.

[3] Huang P., Kim K., Schermer M. (2022). Ethical Issues of Digital Twins for Personalized Health Care Service: Preliminary Mapping Study. J. Med. Internet Res..

[4] Laudisi F., Stolfi C. (2023). Advances in Immunotherapy and Innovative Therapeutic Approaches for Cancer Treatment: Editorial to the Special Issue “State-of-the-Art. Molecular Oncology in Italy”. Int. J. Mol. Sci..

[5] Martinez-Nadal G., Puerta-Alcalde P., Gudiol C., Cardozo C., Albasanz-Puig A., Marco F., Laporte-Amargós J., Moreno-García E., Domingo-Doménech E., Chumbita M. (2020). Inappropriate Empirical Antibiotic Treatment in High-risk Neutropenic Patients With Bacteremia in the Era of Multidrug Resistance. Clin. Infect. Dis..

[6] Garcia-Vidal C., Sanjuan G., Puerta-Alcalde P., Moreno-García E., Soriano A. (2019). Artificial intelligence to support clinical decision-making processes. EBioMedicine.

[7] Lo Presti E., D’Orsi L., De Gaetano A. (2022). A Mathematical Model of In Vitro Cellular Uptake of Zoledronic Acid and Isopentenyl Pyrophosphate Accumulation. Pharmaceutics.

[8] Misra D., Avula V., Wolk D.M., Farag H.A., Li J., Mehta Y.B., Sandhu R., Karunakaran B., Kethireddy S., Zand R. (2021). Early Detection of Septic Shock Onset Using Interpretable Machine Learners. J. Clin. Med..

[9] McCulloch W.S., Pitts W. (1943). A logical calculus of the ideas immanent in nervous activity. Bull. Math. Biophys..

[10] I.—COMPUTING MACHINERY AND INTELLIGENCE|Mind|Oxford Academic n.d. https://academic.oup.com/mind/article/LIX/236/433/986238.

[11] Dobrev D. (2012). A Definition of Artificial Intelligence.

[12] Bahmer A., Gupta D., Effenberger F. (2023). Modern Artificial Neural Networks: Is Evolution Cleverer?. Neural Comput..

[13] Barak O., Tsodyks M. (2023). Mathematical models of learning and what can be learned from them. Curr. Opin. Neurobiol..

[14] Lee M. (2023). Recent Advancements in Deep Learning Using Whole Slide Imaging for Cancer Prognosis. Bioengineering.

[15] Meng Y., Yang Y., Hu M., Zhang Z., Zhou X. (2023). Artificial intelligence-based radiomics in bone tumors: Technical advances and clinical application. Semin. Cancer Biol..

[16] Zeng C., Zhong L., Liu W., Zhang Y., Yu X., Wang X., Zhang R., Kang T., Liao D. (2023). Targeting the Lysosomal Degradation of Rab22a-NeoF1 Fusion Protein for Osteosarcoma Lung Metastasis. Adv. Sci..

[17] Riihimäki M., Thomsen H., Sundquist K., Sundquist J., Hemminki K. (2018). Clinical landscape of cancer metastases. Cancer Med..

[18] Li T., Li Y., Zhu X., He Y., Wu Y., Ying T., Xie Z. (2023). Artificial intelligence in cancer immunotherapy: Applications in neoantigen recognition, antibody design and immunotherapy response prediction. Semin. Cancer Biol..

[19] Hui G., Stefanoudakis D., Zektser Y., Isaacs D.J., Hannigan C., Pantuck A.J., Drakaki A. (2023). Do Cancer Genetics Impact Treatment Decision Making? Immunotherapy and Beyond in the Management of Advanced and Metastatic Urothelial Carcinoma. Curr. Oncol..

[20] Barone B., Calogero A., Scafuri L., Ferro M., Lucarelli G., Di Zazzo E., Sicignano E., Falcone A., Romano L., De Luca L. (2022). Immune Checkpoint Inhibitors as a Neoadjuvant/Adjuvant Treatment of Muscle-Invasive Bladder Cancer: A Systematic Review. Cancers.

[21] Cheng W., Fu D., Xu F., Zhang Z. (2018). Unwrapping the genomic characteristics of urothelial bladder cancer and successes with immune checkpoint blockade therapy. Oncogenesis.

[22] Schumacher T.N., Schreiber R.D. (2015). Neoantigens in cancer immunotherapy. Science.

[23] Lee J.B., Kim H.R., Ha S.-J. (2022). Immune Checkpoint Inhibitors in 10 Years: Contribution of Basic Research and Clinical Application in Cancer Immunotherapy. Immune Netw..

[24] Galon J., Costes A., Sanchez-Cabo F., Kirilovsky A., Mlecnik B., Lagorce-Pagès C., Tosolini M., Camus M., Berger A., Wind P. (2006). Type, density, and location of immune cells within human colorectal tumors predict clinical outcome. Science.

[25] Galon J., Lanzi A. (2020). Immunoscore and its introduction in clinical practice. Q. J. Nucl. Med. Mol. Imaging.

[26] Havel J.J., Chowell D., Chan T.A. (2019). The evolving landscape of biomarkers for checkpoint inhibitor immunotherapy. Nat. Rev. Cancer.

[27] Helmink B.A., Reddy S.M., Gao J., Zhang S., Basar R., Thakur R., Yizhak K., Sade-Feldman M., Blando J., Han G. (2020). B cells and tertiary lymphoid structures promote immunotherapy response. Nature.

[28] Sautès-Fridman C., Petitprez F., Calderaro J., Fridman W.H. (2019). Tertiary lymphoid structures in the era of cancer immunotherapy. Nat. Rev. Cancer.

[29] Colbeck E.J., Ager A., Gallimore A., Jones G.W. (2017). Tertiary Lymphoid Structures in Cancer: Drivers of Antitumor Immunity, Immunosuppression, or Bystander Sentinels in Disease?. Front. Immunol..

[30] Posch F., Silina K., Leibl S., Mündlein A., Moch H., Siebenhüner A., Samaras P., Riedl J., Stotz M., Szkandera J. (2018). Maturation of tertiary lymphoid structures and recurrence of stage II and III colorectal cancer. Oncoimmunology.

[31] Siliņa K., Soltermann A., Attar F.M., Casanova R., Uckeley Z.M., Thut H., Wandres M., Isajevs S., Cheng P., Curioni-Fontecedro A. (2018). Germinal Centers Determine the Prognostic Relevance of Tertiary Lymphoid Structures and Are Impaired by Corticosteroids in Lung Squamous Cell Carcinoma. Cancer Res..

[32] Yarchoan M., Johnson B.A., Lutz E.R., Laheru D.A., Jaffee E.M. (2017). Erratum: Targeting neoantigens to augment antitumour immunity. Nat. Rev. Cancer.

[33] Lee J.H., Kim H., Lee S.H., Ku J.L., Chun J.W., Seo H.Y., Kim S.C., Paik W.H., Ryu J.K., Lee S.K. (2022). Establishment of Patient-Derived Pancreatic Cancer Organoids from Endoscopic Ultrasound-Guided Fine-Needle Aspiration Biopsies. Gut Liver.

[34] Pertseva M., Gao B., Neumeier D., Yermanos A., Reddy S.T. (2021). Applications of Machine and Deep Learning in Adaptive Immunity. Annu. Rev. Chem. Biomol. Eng..

[35] Liu G., Zeng H., Mueller J., Carter B., Wang Z., Schilz J., Horny G., Birnbaum M.E., Ewert S., Gifford D.K. (2020). Antibody complementarity determining region design using high-capacity machine learning. Bioinformatics.

[36] Mason D.M., Friedensohn S., Weber C.R., Jordi C., Wagner B., Meng S.M., Ehling R.A., Bonati L., Dahinden J., Gainza P. (2021). Optimization of therapeutic antibodies by predicting antigen specificity from antibody sequence via deep learning. Nat. Biomed. Eng..

[37] Lim Y.W., Adler A.S., Johnson D.S. (2022). Predicting antibody binders and generating synthetic antibodies using deep learning. mAbs.

[38] Saka K., Kakuzaki T., Metsugi S., Kashiwagi D., Yoshida K., Wada M., Tsunoda H., Teramoto R. (2021). Antibody design using LSTM based deep generative model from phage display library for affinity maturation. Sci. Rep..

[39] Makowski E.K., Kinnunen P.C., Huang J., Wu L., Smith M.D., Wang T., Desai A.A., Streu C.N., Zhang Y., Zupancic J.M. (2022). Co-optimization of therapeutic antibody affinity and specificity using machine learning models that generalize to novel mutational space. Nat. Commun..

[40] Ruffolo J.A., Guerra C., Mahajan S.P., Sulam J., Gray J.J. (2020). Geometric potentials from deep learning improve prediction of CDR H3 loop structures. Bioinformatics.

[41] Ruffolo J.A., Sulam J., Gray J.J. (2022). Antibody structure prediction using interpretable deep learning. Patter.

[42] Akpinaroglu D., Ruffolo J.A., Mahajan S.P., Gray J.J. (2022). Simultaneous prediction of antibody backbone and side-chain conformations with deep learning. PLoS ONE.

[43] Abanades B., Georges G., Bujotzek A., Deane C.M. (2022). ABlooper: Fast accurate antibody CDR loop structure prediction with accuracy estimation. Bioinformatics.

[44] Cohen T., Halfon M., Schneidman-Duhovny D. (2022). NanoNet: Rapid and accurate end-to-end nanobody modeling by deep learning. Front. Immunol..

[45] Lai P.-K. (2022). DeepSCM: An efficient convolutional neural network surrogate model for the screening of therapeutic antibody viscosity. Comput. Struct. Biotechnol. J..

[46] Grinshpun B., Thorsteinson N., Pereira J.N., Rippmann F., Nannemann D., Sood V.D., Fomekong Nanfack Y. (2021). Identifying biophysical assays and in silico properties that enrich for slow clearance in clinical-stage therapeutic antibodies. mAbs.

[47] Wollacott A.M., Xue C., Qin Q., Hua J., Bohnuud T., Viswanathan K., Kolachalama V.B. (2019). Quantifying the nativeness of antibody sequences using long short-term memory networks. Protein Eng. Des. Sel..

[48] Prihoda D., Maamary J., Waight A., Juan V., Fayadat-Dilman L., Svozil D., Bitton D.A. (2022). BioPhi: A platform for antibody design, humanization, and humanness evaluation based on natural antibody repertoires and deep learning. mAbs.

[49] Mitragotri S., Burke P.A., Langer R. (2014). Overcoming the challenges in administering biopharmaceuticals: Formulation and delivery strategies. Nat. Rev. Drug Discov..

[50] Carter P.J., Rajpal A. (2022). Designing antibodies as therapeutics. Cell.

[51] Lipinski C.A., Lombardo F., Dominy B.W., Feeney P.J. (2001). Experimental and computational approaches to estimate solubility and permeability in drug discovery and development settings. Adv. Drug Deliv. Rev..

[52] McDermott J., Sturtevant D., Kathad U., Varma S., Zhou J., Kulkarni A., Biyani N., Schimke C., Reinhold W.C., Elloumi F. (2022). Artificial intelligence platform, RADR®, aids in the discovery of DNA damaging agent for the ultra-rare cancer Atypical Teratoid Rhabdoid Tumors. Front. Drug Discov..

[53] Lai Q., Wu M., Wang R., Lai W., Tao Y., Lu Y., Wang Y., Yu L., Zhang R., Peng Y. (2020). Cryptophycin-55/52 based antibody-drug conjugates: Synthesis, efficacy, and mode of action studies. Eur. J. Med. Chem..

[54] Angell H., Galon J. (2013). From the immune contexture to the Immunoscore: The role of prognostic and predictive immune markers in cancer. Curr. Opin. Immunol..

[55] Koelzer V.H., Gisler A., Hanhart J.C., Griss J., Wagner S.N., Willi N., Cathomas G., Sachs M., Kempf W., Thommen D.S. (2018). Digital image analysis improves precision of PD-L1 scoring in cutaneous melanoma. Histopathology.

[56] Kapil A., Meier A., Zuraw A., Steele K.E., Rebelatto M.C., Schmidt G., Brieu N. (2018). Deep Semi Supervised Generative Learning for Automated Tumor Proportion Scoring on NSCLC Tissue Needle Biopsies. Sci. Rep..

[57] Kapil A., Meier A., Zuraw A., Steele K.E., Rebelatto M.C., Schmidt G., Brieu N. (2022). Predicting Tumor Mutational Burden From Lung Adenocarcinoma Histopathological Images Using Deep Learning. Front. Oncol..

[58] He B., Dong D., She Y., Zhou C., Fang M., Zhu Y., Zhang H., Huang Z., Jiang T., Tian J. (2020). Predicting response to immunotherapy in advanced non-small-cell lung cancer using tumor mutational burden radiomic biomarker. J. Immunother. Cancer.

[59] Huang K., Lin B., Liu J., Liu Y., Li J., Tian G., Yang J. (2022). Predicting colorectal cancer tumor mutational burden from histopathological images and clinical information using multi-modal deep learning. Bioinformatics.

[60] Topalian S.L., Taube J.M., Anders R.A., Pardoll D.M. (2016). Mechanism-driven biomarkers to guide immune checkpoint blockade in cancer therapy. Nat. Rev. Cancer.

[61] Wu J., Liu C., Liu X., Sun W., Li L., Gao N., Zhang Y., Yang X., Zhang J., Wang H. (2022). Artificial intelligence-assisted system for precision diagnosis of PD-L1 expression in non-small cell lung cancer. Mod. Pathol..

[62] Huang Z., Chen L., Lv L., Fu C.C., Jin Y., Zheng Q., Wang B., Ye Q., Fang Q., Li Y. (2022). A new AI-assisted scoring system for PD-L1 expression in, NSCLC. Comput. Methods Programs Biomed..

[63] Pollack M.H., Betof A., Dearden H., Rapazzo K., Valentine I., Brohl A.S., Ancell K.K., Long G.V., Menzies A.M., Eroglu Z. (2018). Safety of resuming anti-PD-1 in patients with immune-related adverse events (irAEs) during combined anti-CTLA-4 and anti-PD1 in metastatic melanoma. Ann. Oncol..

[64] Michot J.M., Bigenwald C., Champiat S., Collins M., Carbonnel F., Postel-Vinay S., Berdelou A., Varga A., Bahleda R., Hollebecque A. (2016). Immune-related adverse events with immune checkpoint blockade: A comprehensive review. Eur. J. Cancer.

[65] Tran K.A., Kondrashova O., Bradley A., Williams E.D., Pearson J.V., Waddell N. (2021). Deep learning in cancer diagnosis, prognosis and treatment selection. Genome Med..

[66] Wang Q., Xu R. (2019). Immunotherapy-related adverse events (irAEs): Extraction from FDA drug labels and comparative analysis. JAMIA Open.

[67] Xie J., Luo X., Deng X., Tang Y., Tian W., Cheng H., Zhang J., Zou Y., Guo Z., Xie X. (2023). Advances in artificial intelligence to predict cancer immunotherapy efficacy. Front. Immunol..

[68] Chan T.A., Yarchoan M., Jaffee E., Swanton C., Quezada S.A., Stenzinger A., Peters S. (2019). Development of tumor mutation burden as an immunotherapy biomarker: Utility for the oncology clinic. Ann. Oncol..

[69] Kather J.N., Pearson A.T., Halama N., Jäger D., Krause J., Loosen S.H., Marx A., Boor P., Tacke F., Neumann U.P. (2019). Deep learning can predict microsatellite instability directly from histology in gastrointestinal cancer. Nat. Med..

[70] Echle A., Grabsch H.I., Quirke P., van den Brandt P.A., West N.P., Hutchins G.G., Heij L.R., Tan X., Richman S.D., Krause J. (2020). Clinical-Grade Detection of Microsatellite Instability in Colorectal Tumors by Deep Learning. Gastroenterology.

[71] Yamashita R., Long J., Longacre T., Peng L., Berry G., Martin B., Higgins J., Rubin D.L., Shen J. (2021). Deep learning model for the prediction of microsatellite instability in colorectal cancer: A diagnostic study. Lancet Oncol..

[72] Xie F., Zhang J., Wang J., Reuben A., Xu W., Yi X., Varn F.S., Ye Y., Cheng J., Yu M. (2020). Multifactorial deep learning reveals pan-cancer genomic tumor clusters with distinct immunogenomic landscape and response to immunotherapy. Clin. Cancer Res..

[73] Van Allen E.M., Miao D., Schilling B., Shukla S.A., Blank C., Zimmer L., Sucker A., Hillen U., Geukes Foppen M.H., Goldinger S.M. (2015). Genomic correlates of response to CTLA-4 blockade in metastatic melanoma. Science.

[74] Xie J., Zou Y., Ye F., Zhao W., Xie X., Ou X., Xie X., Wei W. (2022). A Novel Platelet-Related Gene Signature for Predicting the Prognosis of Triple-Negative Breast Cancer. Front. Cell Dev. Biol..

[75] Yadav M., Jhunjhunwala S., Phung Q.T., Lupardus P., Tanguay J., Bumbaca S., Franci C., Cheung T.K., Fritsche J., Weinschenk T. (2014). Predicting immunogenic tumour mutations by combining mass spectrometry and exome sequencing. Nature.

[76] Mo X., Tang C., Niu Q., Ma T., Du Y., Fu H. (2019). HTiP: High-Throughput Immunomodulator Phenotypic Screening Platform to Reveal IAP Antagonists as Anti-cancer Immune Enhancers. Cell Chem. Biol..

[77] Lo Presti E., Mocciaro F., Mitri R.D., Corsale A.M., Di Simone M., Vieni S., Scibetta N., Unti E., Dieli F., Meraviglia S. (2020). Analysis of colon-infiltrating γδ T cells in chronic inflammatory bowel disease and in colitis-associated cancer. J. Leukoc. Biol..

[78] Lo Presti E., Dieli F., Fourniè J.J., Meraviglia S. (2020). Deciphering human γδ T cell response in cancer: Lessons from tumor-infiltrating γδ T cells. Immunol. Rev..

[79] Scholler N., Perbost R., Locke F.L., Jain M.D., Turcan S., Danan C., Chang E.C., Neelapu S.S., Miklos D.B., Jacobson C.A. (2022). Tumor immune contexture is a determinant of anti-CD19 CAR T cell efficacy in large B cell lymphoma. Nat. Med..

[80] Menden K., Marouf M., Oller S., Dalmia A., Magruder D.S., Kloiber K., Heutink P., Bonn S. (2020). Deep learning-based cell composition analysis from tissue expression profiles. Sci. Adv..

[81] Chakravarthy A., Furness A., Joshi K., Ghorani E., Ford K., Ward M.J., King E.V., Lechner M., Marafioti T., Quezada S.A. (2018). Pan-cancer deconvolution of tumour composition using DNA methylation. Nat. Commun..

[82] Zaitsev A., Chelushkin M., Dyikanov D., Cheremushkin I., Shpak B., Nomie K., Zyrin V., Nuzhdina E., Lozinsky Y., Zotova A. (2022). Precise reconstruction of the TME using bulk RNA-seq and a machine learning algorithm trained on artificial transcriptomes. Cancer Cell.

[83] Saltz J., Gupta R., Hou L., Kurc T., Singh P., Nguyen V., Samaras D., Shroyer K.R., Zhao T., Batiste R. (2018). Spatial Organization and Molecular Correlation of Tumor-Infiltrating Lymphocytes Using Deep Learning on Pathology Images. Cell Rep..

[84] Rakhlin A., Tiulpin A., Shvets A.A., Kalinin A.A., Iglovikov V.I., Nikolenko S. Breast Tumor Cellularity Assessment Using. Deep Neural Networks. Proceedings of the 2019 IEEE/CVF International Conference on Computer Vision Workshop (ICCVW).

[85] Choi H., Na K.J., Koh J., Kim Y.T. (2022). Abstract 5131: Deep learning-based tumor microenvironment cell types mapping from H&E images of lung adenocarcinoma using spatial transcriptomic data. Cancer Res..

[86] Kong J., Ha D., Lee J., Kim I., Park M., Im S.H., Shin K., Kim S. (2022). Network-based machine learning approach to predict immunotherapy response in cancer patients. Nat. Commun..

[87] Vanguri R.S., Luo J., Aukerman A.T., Egger J.V., Fong C.J., Horvat N., Pagano A., Araujo-Filho J.D.A.B., Geneslaw L., Rizvi H. (2022). Multimodal integration of radiology, pathology and genomics for prediction of response to PD-(L)1 blockade in patients with non-small cell lung cancer. Nat. Cancer.

[88] Chowell D., Yoo S.K., Valero C., Pastore A., Krishna C., Lee M., Hoen D., Shi H., Kelly D.W., Patel N. (2022). Improved prediction of immune checkpoint blockade efficacy across multiple cancer types. Nat. Biotechnol..

[89] Razdan S., Sharma S. (2022). Internet of Medical Things (IoMT): Overview, Emerging Technologies, and Case Studies. IETE Tech. Rev..

[90] Moztarzadeh O., Jamshidi M., Sargolzaei S., Jamshidi A., Baghalipour N., Malekzadeh Moghani M., Hauer L. (2023). Metaverse and Healthcare: Machine Learning-Enabled Digital Twins of Cancer. Bioengineering.

[91] Kovatchev B. (2018). The year of transition from research to clinical practice. Nat. Rev. Endocrinol..

[92] Susilo M.E., Li C.C., Gadkar K., Hernandez G., Huw L.Y., Jin J.Y., Yin S., Wei M.C., Ramanujan S., Hosseini I. (2023). Systems-based digital twins to help characterize clinical dose-response propose predictive biomarkers in a Phase I study of bispecific antibody mosunetuzumab in, NHL. Clin. Transl. Sci..

[93] Sarris A.L., Sidiropoulos E., Paraskevopoulos E., Bamidis P. (2023). Towards a Digital Twin in Human Brain: Brain Tumor Detection Using K-Means. Stud. Health Technol. Inform..

[94] Keller J., Lindenmeyer A., Blattmann M., Gaebel J., Schneider D., Neumuth T., Franke S. (2023). Using Digital Twins to Support Multiple Stages of the Patient Journey. Stud. Health Technol. Inform..

[95] Lareyre F., Adam C., Carrier M., Raffort J. (2020). Using Digital Twins for Precision Medicine in Vascular Surgery. Ann. Vasc. Surg..

[96] A Semi-Active Human Digital Twin Model for Detecting Severity of Carotid Stenoses from Head Vibration-A Coupled Computational Mechanics and Computer Vision Method-PubMed n.d. https://pubmed.ncbi.nlm.nih.gov/30648344/.

[97] Winter P.D., Chico T.J.A. (2023). Using the Non-Adoption, Abandonment, Scale-Up, Spread, and Sustainability (NASSS) Framework to Identify Barriers and Facilitators for the Implementation of Digital Twins in Cardiovascular Medicine. Sensors.

[98] Sun T., He X., Li Z. (2023). Digital twin in healthcare: Recent updates and challenges. Digit. Health.

