# Supplementary material for: Recent Advances in Artificial Intelligence to Improve Immunotherapy and the Use of Digital Twins to Identify Prognosis of Patients with Solid Tumors
Source: Int J Mol Sci. 2024 Oct 29;25(21):11588. doi: 10.3390/ijms252111588 (PMC11546512; doi:10.3390/ijms252111588)

Figure S1: The flowchart provides a brief overview of how the information summarized in our narrative review was gathered. The boxes show the number of records identified, included, and excluded, along with the reasons for exclusion.

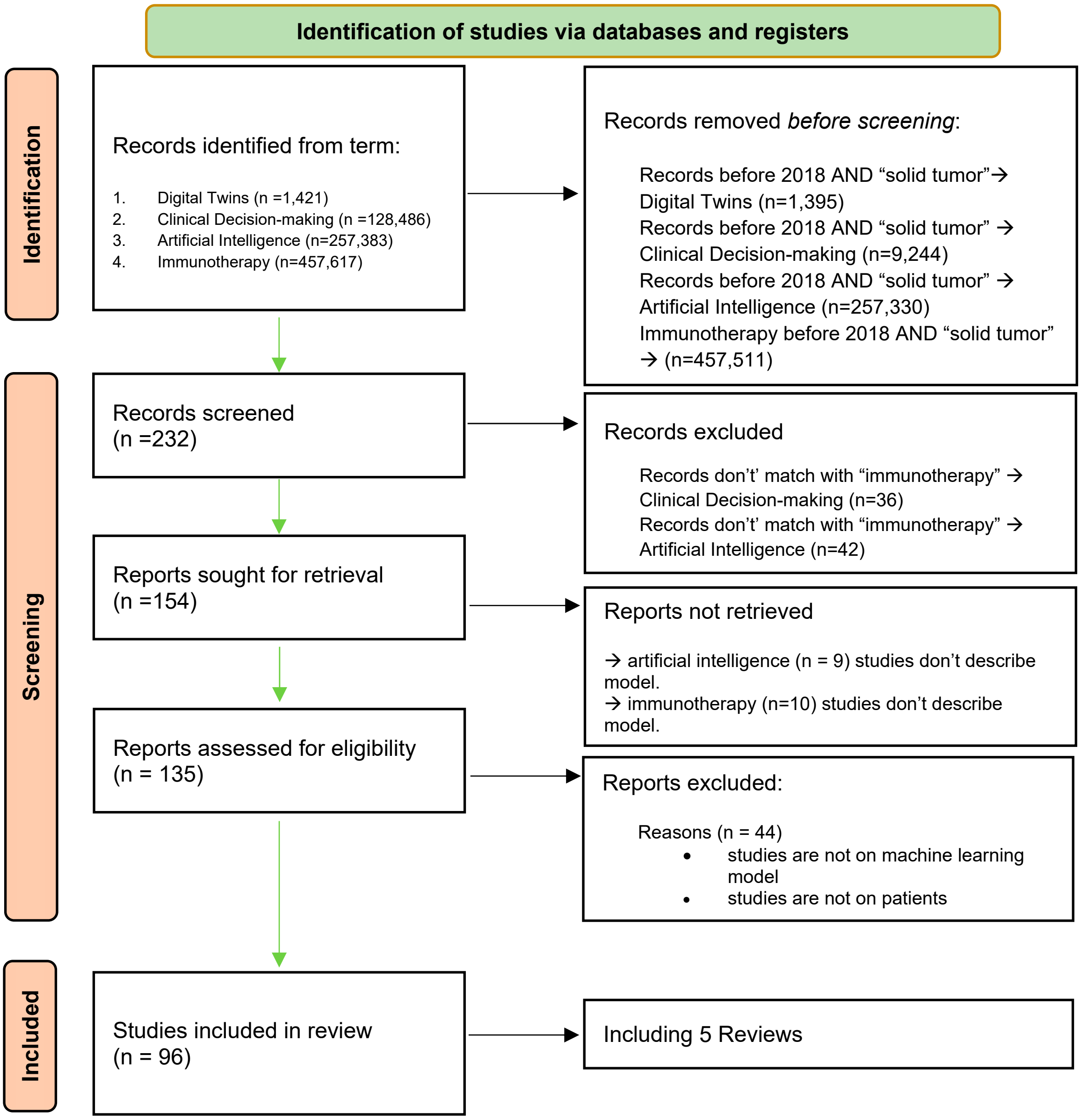

Supplement: Supplementary file 1 [file ijms-25-11588-s001.zip › ijms-3173679-supplementary.pdf]
